# Diurnal variation of human tear meniscus volume measured with tear strip meniscometry self-examination

**DOI:** 10.1371/journal.pone.0215922

**Published:** 2019-04-23

**Authors:** Masahiko Ayaki, Naoko Tachi, Yoshihiro Hashimoto, Motoko Kawashima, Kazuo Tsubota, Kazuno Negishi

**Affiliations:** 1 Department of Ophthalmology, Keio University School of Medicine, Tokyo, Japan; 2 Otake Clinic Moon View Eye Center, Yamato, Japan; 3 Shiseikai Toyama Hospital Eye Center, Toyama, Japan; University of California Berkeley, UNITED STATES

## Abstract

**Purpose:**

Tear secretion is critical for maintenance of healthy ocular surface and vision. Most of normal subjects and dry eye patients feel worsening of ocular dryness in the afternoon, however, diurnal variation of tear meniscus volume has not been directly measured because there was no appropriate method. We used a simple, non-invasive technique, tear strip meniscometry (SM) by self-examination. Previous investigations indicated the values of SM correlated strongly with those of the Schirmer test, tests of tear break up time, and tear meniscus height measurement by anterior optical coherence tomography. The purpose of this study was to capture diurnal variation of aqueous availability at the tear meniscus by measuring wetted length using SM through self-examination.

**Methods:**

Thirty-six medical personnel (mean age; 35.7 years) participated and SM self-examination was performed using a mirror seven times a day. The strip is applied for 5 seconds to the lateral side of the lower lid tear meniscus without touching the ocular surface.

**Results:**

The measured SM value was the highest upon awakening (4.44 ± 3.14 mm) and gradually decreased in the evening; 3.81 ± 3.12 at 9:00, 3.31 ± 2.72* at 12:00, 2.89 ± 1.88* at 15:00, 2.92 ± 1.87* at 18:00, 2.78 ± 1.85* at 21:00, and 2.89 ± 1.75* at bedtime with statistical significance compared to the value upon awakening (*P < 0.05, Dunnett’s multiple comparison test). Proportion of number of subjects with low SM value (< 4 mm) to total number of subjects was 52.8% upon awakening and 83.3% at 21:00, and gradually increased toward evening.

**Conclusion:**

Our results could identify diurnal variation of tear meniscus volume in the general population.

## Introduction

Tear secretion from the lacrimal glands is controlled by the autonomic nervous system, predominantly by parasympathetic input [1]. Tears cover and protect the ocular surface, and stability of the ocular surface is critical for ocular health and vision. Tear secretion deficiency is closely related to dry eye disease (DED) and is diagnosed by a deficit in tear secretion, corneal damage, and related ocular symptoms. It is most prevalent in middle-aged women and often accompanied by decreased quality of life, depression, and sleep disorder [2,3]. Many people feel worse ocular symptoms, including dryness and discomfort, in the evening [4–6]. However, the etiology of this is elusive. The ocular surface can be damaged by blue light [7–9] in the evening, because modern workplaces and homes are equipped with blue light-rich lighting, and self-luminous devices are used throughout the day [10].

Numerous clinical methods have been used to evaluate tear secretion, but most are invasive and require qualified personnel and special instruments or staining. The Schirmer test is the gold standard for tear quantity assessment and is performed by inserting a strip of filter paper into the lower conjunctival sac to absorb tear produced by basic and reflex tearing. Its disadvantages include irritation, pain, and conjunctival epithelial damage. It is also time consuming; measurements can take as long as 5 minutes. Furthermore, it is not suitable for repeated examinations on the same day due to ocular surface damage caused by the filter paper.

**Fig 1:**
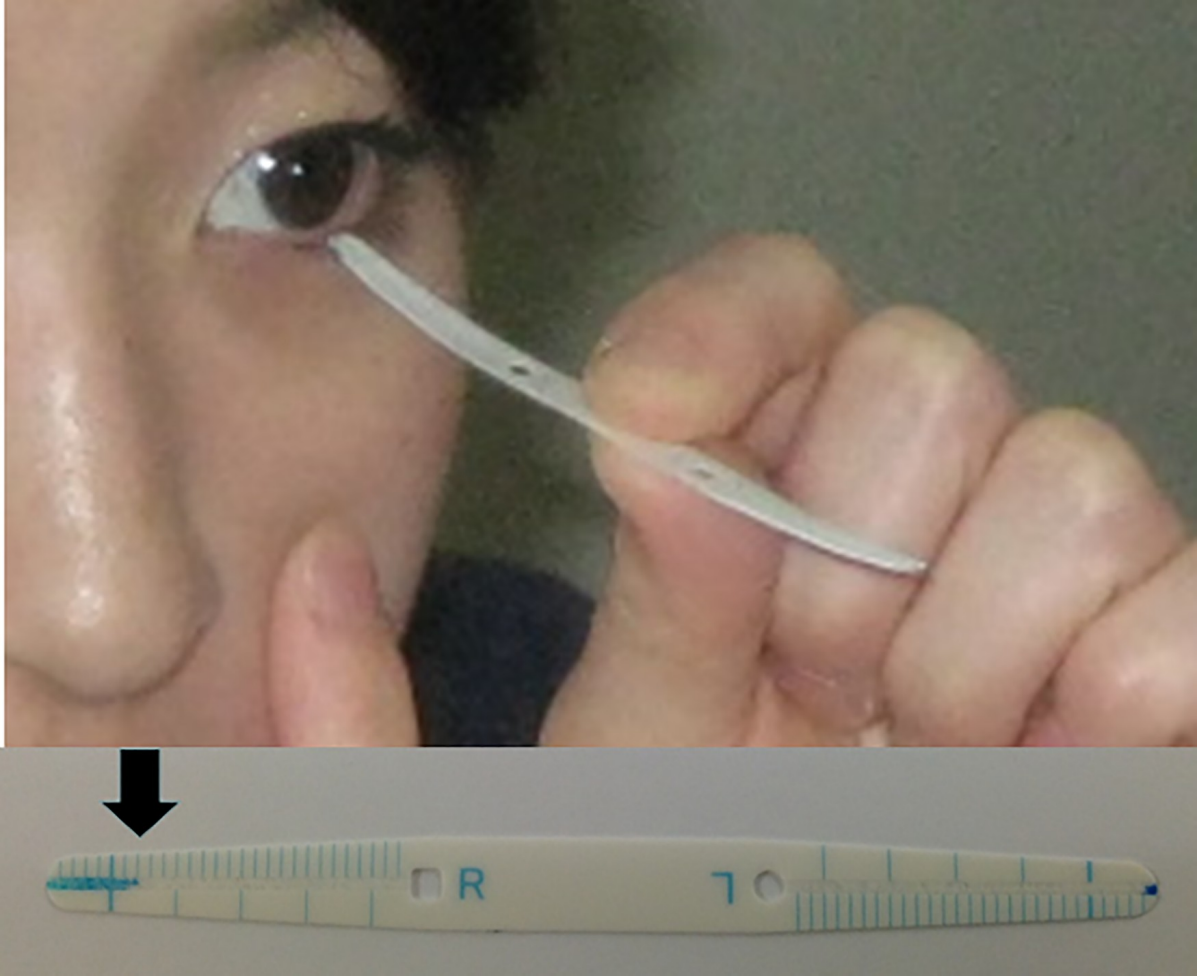
Strip meniscometry self-examination technique. Strip meniscometry (SM) self-examination can be performed using a mirror (top). The length of the blue line (arrow) is regarded as the SM value in the eye (bottom).

Diurnal variation studies of tear parameters are scarce, probably because of limitations of resources or techniques. Diurnal variation has previously been examined using five optical coherence tomography measurements (from 9:00 to 21:00) [11] and using four Schirmer test measurements during hospitalization [12]. Tear strip meniscometry (SM) was recently introduced to be a non-invasive, fast, and simple test for measuring aqueous availability at the tear meniscus [13–19] (Fig 1). The structure and composition of meniscometry strips and the technique of SM have been described in detail previously [13,14]. Briefly, the meniscometry strips contain nitrocellulose membranes with a pore size of 8 μm (width, 3 mm; height, 45 mm). Each strip has natural blue dye 1 printed near its tip. The membrane filter paper strips are coated on both sides with a hydrophobic polyether masking membrane and are press processed to eliminate the polyether masking film centrally, to create a central ditch that is 0.5 mm wide and 100 μm deep. When the strip is immersed in tears, the tears enter the ditch, change to blue color when they contact the blue dye near the tip of the strip, and remain in the ditch without spreading sideways due to the hydrophobic masking membrane. SM self-examination can be performed using a mirror (Fig 1). SM was first described at 2006 and proven to have statistically significant linear correlation with the Schirmer test value, tear break-up time, corneal staining score, and tear meniscus height measurement by anterior optical coherence tomography [13,14]. The *in vivo* and *in vitro* reproducibility, sensitivity, and specificity were sufficiently evaluated [13] and SM has been used in many studies [14–19]. The purpose of this study was to capture diurnal variation of aqueous availability at the tear meniscus by measuring wetted length using SM through self-examination with seven measurements taken upon awakening to immediately before bedtime.

## Materials and methods

This study was approved by an Ethics Committee of Shinseikai Toyama Hospital (the hospital within which the work was undertaken) (approval number; 150110–1), and it conforms to the provisions of the 1995 Declaration of Helsinki (as revised in Edinburgh, 2000). Written informed consent was obtained from every participant and the study was performed in accordance with approved protocol. The participants were 36 ophthalmic technicians and other health professionals (23 females; mean age, 35.7 ± 10.5 years). Normal vision, systemic health, no ocular comorbidity of all participants was confirmed by ophthalmologists and annual health check-ups conducted at each participant’s workplace. None of the participants used contact lenses, did shift work, or had circadian rhythm disorders according to the validated and sophisticated sleep questionnaire; the Pittsburgh Sleep Quality Index [20].

Participants were asked to perform SM self-examination using a mirror in both eyes at the following times: upon awakening, 9:00, 12:00, 15:00, 18:00, 21:00, and bedtime. Measurements were performed in a medical facility from 9:00 until 18:00 and at each participant’s home upon awakening, at 21:00, and at bedtime. To obtain the wetted length, the strips were inserted for 5 seconds into the lateral side of the lower lid tear meniscus without touching the ocular surface. Certified orthoptists with national licensure provided the participants with instructions for and training in the self-meniscometry technique, and the participants also received an instruction manual. All participants practiced the technique at least twice before the experiment began, and followed the same protocol. Room temperature and humidity were kept at 21–24 degrees centigrade and 30–60%, respectively, during measurement inside the medical facility to control for evaporative effects [21]. The other measurements were performed inside the participants’ homes at comfortable levels of air conditioning.

The values obtained for the right eye of each participant were analyzed. Outlier values were not excluded, even though there was a wide variation in the data among participants. Where appropriate, data are reported as mean ± standard deviation (SD). Mean value at each time was compared to that upon awakening with Dunnett’s multiple comparison test. Analyses were performed using StatFlex (Atech, Osaka, Japan). P values less than 0.05 were considered significant.

## Results

**Fig 2:**
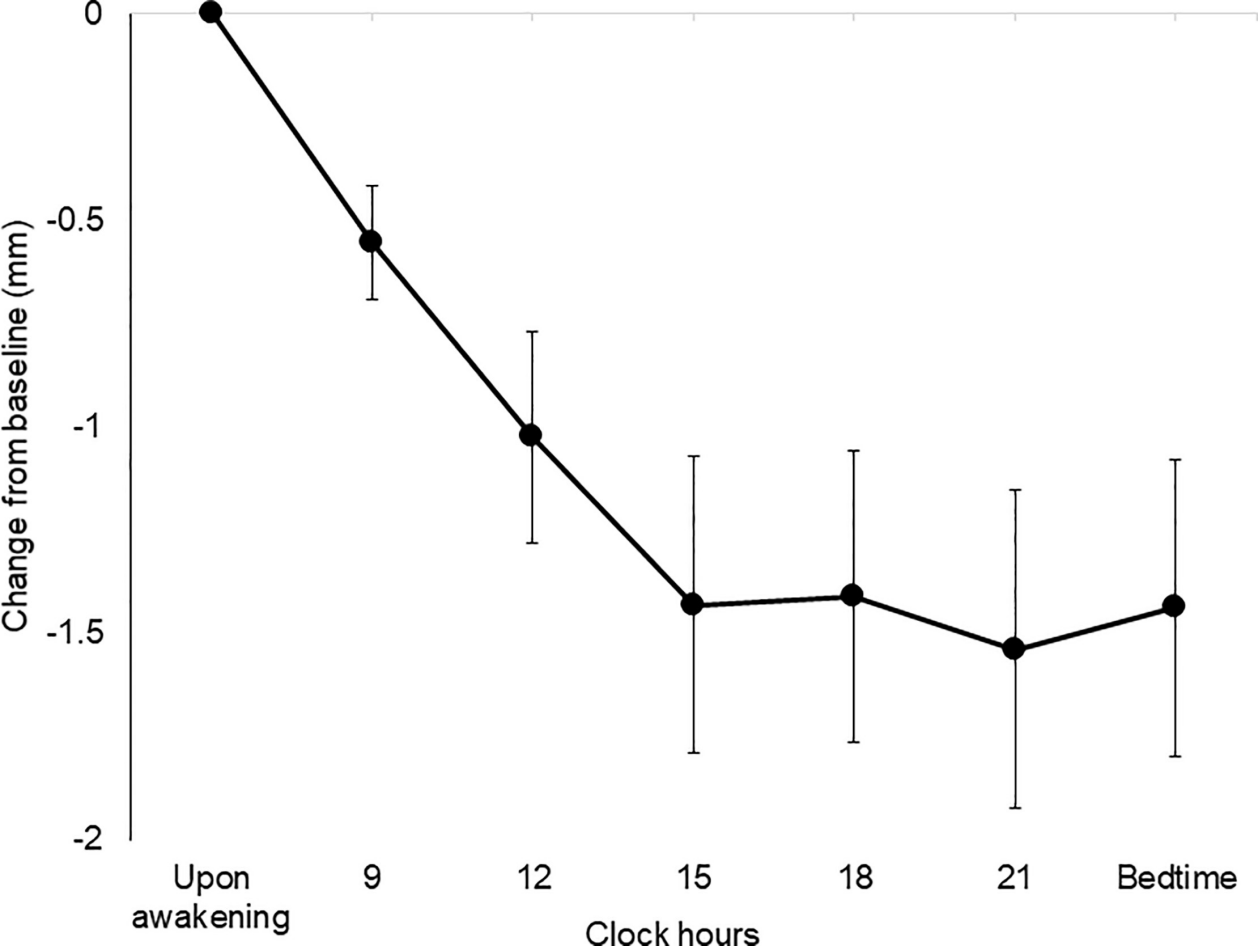
Change from baseline of strip meniscometry.

**Fig 3:**
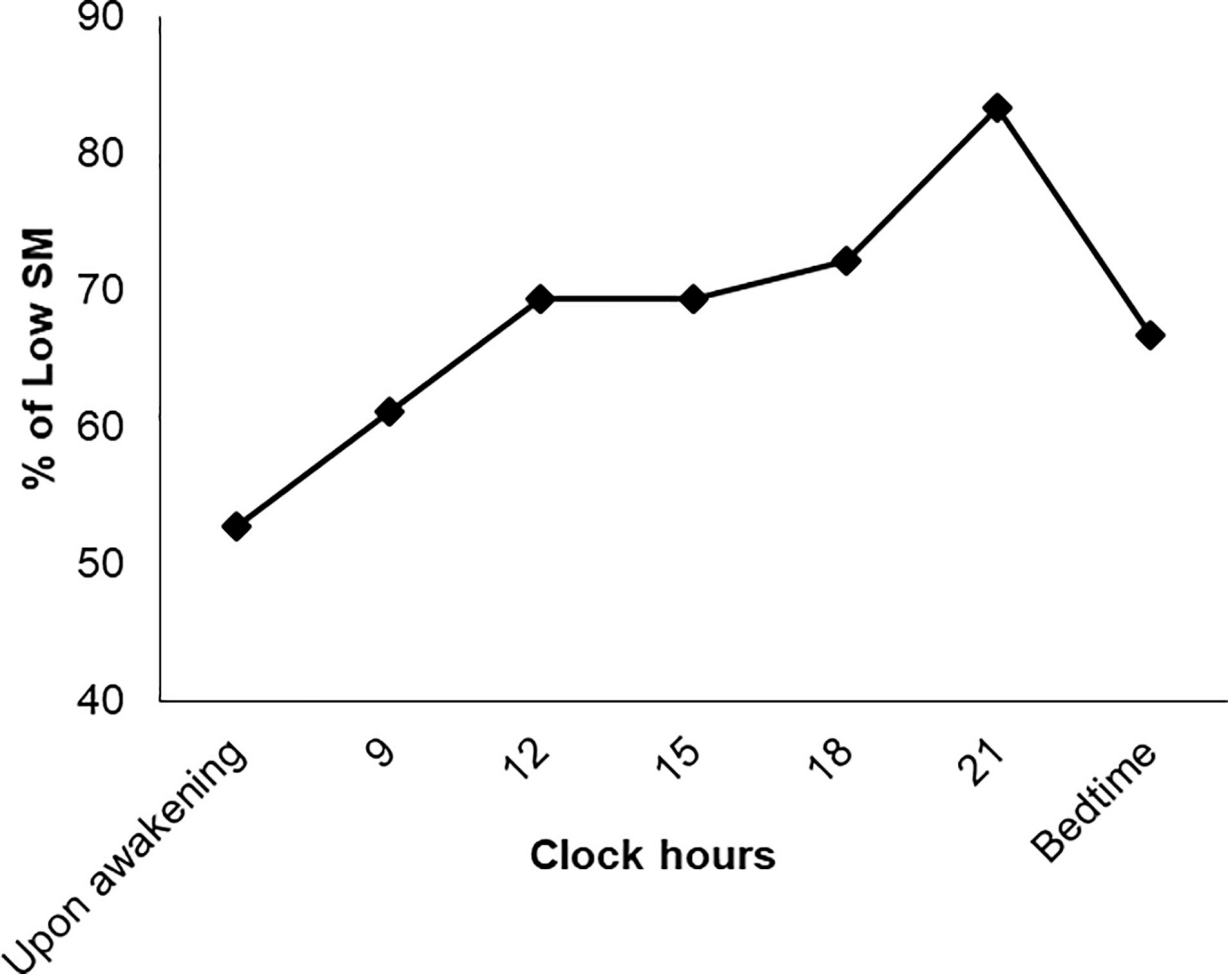
Diurnal variation of the proportion of number of subjects with low strip meniscometry (SM) value (< 4 mm). The proportion was 52.8% upon awakening and 83.3% at 21:00, respectively, and gradually increased toward evening.

All participants successfully performed examinations without complications such as pain or conjunctival injection. The mean, minimum, and maximum SM values (mm) are shown in Table 1 (S1 Fig and S1 Table). Mean SM value at 12:00, 15:00, 18:00, 21:00, and at bedtime were statistically significantly different (*P* < 0.05) compared to the mean SM value upon awakening. To further clarify the diurnal variation, change from baseline of SM upon awakening is shown in Fig 2. The proportion was calculated by dividing the number of subjects with a low SM value (< 4 mm) with the total number of subjects and it was 52.8% upon awakening and 83.3% at 21:00, and gradually increased toward evening (Fig 3). When compared to the proportion at awakening, proportions measured at all time points were not significantly different: *P* = 0.475 at 9:00, *P* = 0.147 at 12:00 and 15:00, *P* = 0.088 at 18:00, *P* = 0.054 at 21:00, and *P* = 0.230 at bedtime (vs upon awakening, chi-squared test), respectively. The maximum value did not exceed 4mm at any time point in nine participants (40.0%).

**Table 1:** Results of tear strip meniscometry.

Time	Upon awakening	9:00	12:00	15:00	18:00	21:00	Bedtime
N	36	36	36	36	36	36	36
Mean± SD (mm)	4.44 ± 3.14	3.81 ± 3.12	3.31 ± 2.72	2.89 ± 1.88	2.92 ± 1.87	2.78 ± 1.85	2.89 ± 1.75
Maximum (mm)	13	15	13	9	8	10	8
Minimum (mm)	1	1	1	1	1	1	1

## Discussion

We obtained SM measurements seven times a day using a non-invasive method and our results clearly demonstrated diurnal variation in SM values. Our results, which show that SM value is highest upon awakening and that it decreases in the afternoon and evening, could account for these commonly observed clinical symptoms. High SM values upon awakening might be associated with the turnover and defense mechanisms of tear film during eye closure for sleep [22]. Even for people with high SM value in the morning, tear film status may decline to abnormal levels in the afternoon, and these people might suffer from dryness. We believe that our results reliably indicate changes in tear meniscus volume since medical personnel who are familiar with ophthalmic examinations performed all the measurements. Although further studies are warranted to verify our results with a gold standard method such as the Schirmer’s test with repeated measurements and correlations with symptoms, we believe our study successfully identified diurnal changes in aqueous availability at the tear meniscus in the general population as can be seen in S2 Fig,

Based on our results, DED care in accordance with diurnal variation of tear meniscus volume could be useful. Special care should be taken to protect the ocular surface from afternoon dryness ― for example, application of eyedrops, maintenance of eyelid hygiene, and environmental accommodation of humidity. Our results may provide new insight into understanding and managing DED.

It should be noted that the measured SM value does not directly quantify tear secretion. Tear meniscus volume is heavily dependent on the anatomy of the eyelids and the fluid volume in the fornix. Huang et al. [23] showed that tear meniscus recovers over 70% of fluid volume from the fornix within 3 seconds without blinking in healthy subjects. Therefore, measured SM values represent a combination of freshly secreted tears from the lacrimal gland and tears stored in the fornix that could have been secreted some time earlier. Eliminating the tear reservoir in the fornix would enable more accurate measurement of tear secretion. Using the geometry of the strip and the porosity of the wetted region of the strip [13], tear meniscus volume can be calculated [24], although this was not done in this study.

SM self-examination is a fast, simple and non-invasive method. It can be repeated throughout a single day, so it can be performed from early morning to late night, and it can be done at home because it does not require a technician or special instruments. Although the Schirmer test is the gold standard method for assessing tear production, it could disturb the ocular surface if used seven times throughout a single day, resulting in abnormal tear secretion. There is a scarcity of reports about diurnal variations in tear availability, and this study proposes to use a technique that may facilitate future studies. SM is as easy as contact lens insertion, and non-specialists can safely perform the technique. The present study was carried out with medical personnel as participants, and was conducted predominantly during office hours in a medical facility. This approach was used in order to minimize potential errors through controlling the study environment, the participants’ activities and other factors. It is useful for chronobiological studies as it enables continuous observation of the activity of the parasympathetic nervous system in the eye. Disadvantages include incomplete insertion of the strip and contact with the eyeball, resulting in insufficient absorption of basic tear fluid and excessive absorption of reflex tear fluid, respectively. According to a previous investigation [13,14], the cut-off value for diagnosis of dry eye by SM was approximately 3–4 mm. However, our results may have underestimated SM values compared with results obtained by methods that require a technician. Low SM values could be due to tear deficiency or insufficient absorption of tear meniscus. Possible factors contributing to the wetted length measured with SM could be reflex tearing, conjunctivochalasis [23], blinking [25,26], and room humidity [21]. SM self-examination may become a useful self-diagnostic tool for use at home by the general population after its accuracy and stability are improved. Like the Schirmer test, which has been used for over 100 years [26], SM could be improved. First, a wider central ditch for absorption could facilitate more stable and efficient absorption. Second, the site of strip insertion, eye position during strip insertion, and head position during strip insertion could be changed, as this might facilitate easier and more effective measurement. Third, reducing the cost could encourage more widespread use and testing by SM because the currently used strips are more expensive than the filter paper used for the Schirmer test. In addition, and very importantly, accumulation of clinical data will contribute to more accurate, stable and standardized application of the technique. Furthermore, developing an organized self-examination kit containing strips, a mirror and an instruction manual might be helpful for facilitating effective use of SM by the general population, because organized saliva, urine, hair and mucosa testing kits are commercially available and have become popular for self-examination.

Limitations of the present study are as follows. SM is a new technique that requires further investigation before it can be considered a standard method for research and clinical applications. In addition to secretion, tear meniscus volume is affected by tear drainage, reflex tears, blinking, conjunctivochalasis and other ocular surface disorders, and these factors should be adjusted for when standardizing SM. Tear secretion may be abnormal in DED, Sjogren’s syndrome, and other systemic disorders; future research should assess whether the diurnal variation of tear secretion is preserved in those conditions. Although we reviewed the results of the participants’ annual health checks and examined the participants’ sleep quality with a validated questionnaire to exclude circadian rhythm disorder, a fundamental limitation of our study is the lack of data on participants’ signs and symptoms of dry eye. Therefore, to further improve our understanding of diurnal variation in signs and symptoms of dry eye, conducting studies with corneal examinations and assessment of symptoms at each time point, including vital staining, tear break-up time, strip meniscometry and optical coherence tomography, would be valuable.

## Supporting information

S1 Table The raw data of the subjects. (XLS)

S1 Fig Diurnal variation of wetted length measured with strip meniscometry (mean and standard deviation). The mean SM value was significantly different at 12:00 (*P* < 0.05), 15:00 (*P* < 0.01), 18:00 (*P* < 0.01), 21:00 (*P* < 0.01), and bedtime (*P* < 0.01) compared to the mean value upon awakening using Dunnett’s multiple comparison test. *P < 0.05
(TIF)

S2 Fig Diurnal variation of wetted length measured with strip meniscometry (raw data of all participants). Wetted length measured with strip meniscometry of all participants. Although the numbers of data lines may not seem to match the participant numbers, most of lines are overlapped since the measured values ranged from 1 to 3 mm in the majority of cases.
(TIF)
