# Supplementary figures and images for: Diurnal variation of human tear meniscus volume measured with tear strip meniscometry self-examination
Source: PLoS One. 2019 Apr 23;14(4):e0215922. doi: 10.1371/journal.pone.0215922 (PMC6478337; doi:10.1371/journal.pone.0215922)

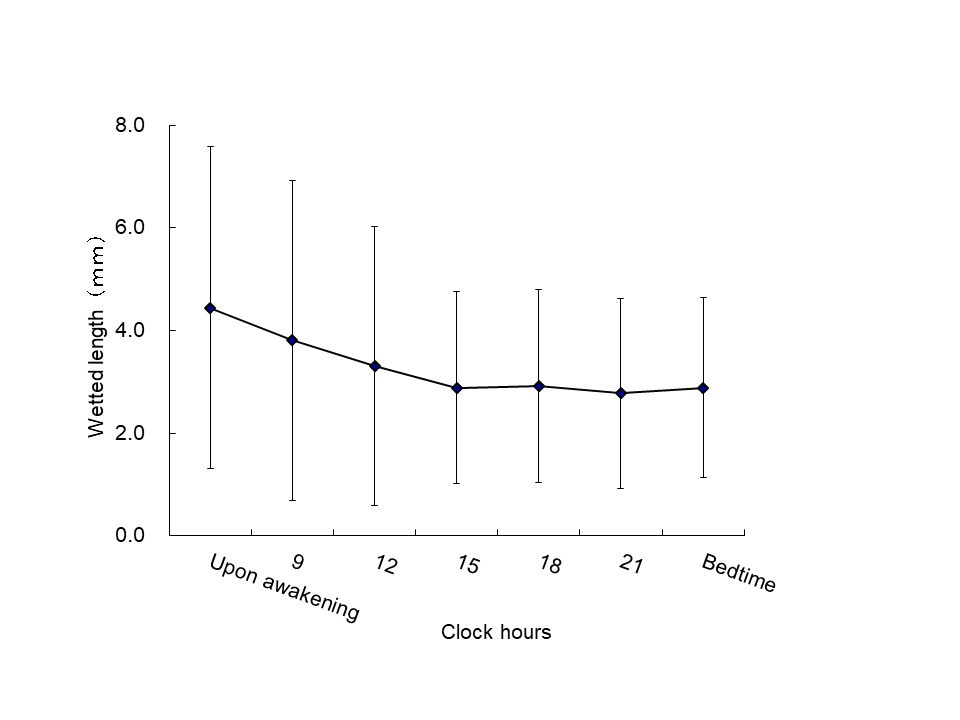

Supplement: S1 Fig — The mean SM value was significantly different at 12:00 (P < 0.05), 15:00 (P < 0.01), 18:00 (P < 0.01), 21:00 (P < 0.01), and bedtime (P < 0.01) compared to the mean value upon awakening using Dunnett’s multiple comparison test. *P < 0.05 (TIF) [file pone.0215922.s002.tif]

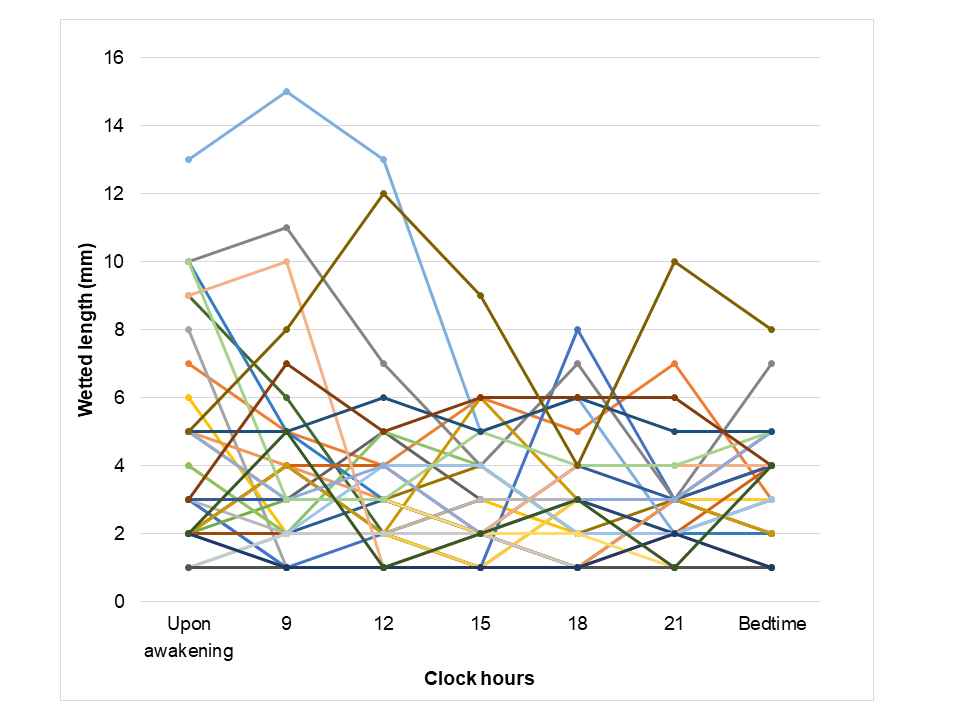

Supplement: S2 Fig — Wetted length measured with strip meniscometry of all participants. Although the numbers of data lines may not seem to match the participant numbers, most of lines are overlapped since the measured values ranged from 1 to 3 mm in the majority of cases. (TIF) [file pone.0215922.s003.tif]
